# Beyond Window Rainbows: Collecting Children’s Culture in the COVID Crisis

**DOI:** 10.1177/1550190620980836

**Published:** 2021-06

**Authors:** Monica Eileen Patterson, Rebecca Friend

**Affiliations:** 1 Carleton University, Ottawa, ON, Canada

**Keywords:** museum, subject focus, collections, ethics, collections, community, research and topics, diversity, equity, inclusion, social engagement, underrepresentation, COVID-19

## Abstract

As COVID-19 dramatically alters the museum sector, museums and archives are implementing collection initiatives that will have tremendous influence over how the pandemic is understood and remembered. As collections experts, museums are leading the charge to document, collect, and interpret our current circumstances as they unfold in real time, relying more than ever on public participation and crowd-sourcing. A key development in such rapid-response collecting has been the interest in and solicitation of contributions that document the current crisis. Yet, initiatives that target young people remain few and far between, and often reproduce romanticized and reified understandings of children and childhood that reflect a longer history of excluding children’s voices from museum collections and society at large. This paper will explore museums’ collection of children’s culture in various forms with attention to the epistemological and ethical challenges that such initiatives entail. We argue that children are crucial citizens whose knowledge, perspectives, and experiences must be collected and preserved during this historic moment and beyond, in ways that attend to the particular circumstances they face as multiply marginalized museum constituents and members of society.

## Introduction

It has become cliché to note that current times are unprecedented. Across the world, people are facing serious challenges to their health, happiness, and well-being as a result of the COVID-19 virus and the measures taken to stop its spread. As a result of the still-unfolding pandemic, many public institutions remain closed indefinitely, while some have reopened with limited capacity. Despite the many challenges they face, museums have shown institutional leadership in these tumultuous times, offering a wide range of enriching online content for children and adults. Museums have created platforms and activities for public engagement, community building, education, personal development, and entertainment. As collections experts, they are also leading the charge to document, collect, and reflect on our current circumstances as they unfold in real time, relying more than ever on public participation and crowd-sourcing. But a key demographic has been largely overlooked in these collecting initiatives. While there has been a proliferation of materials and programming *for* children, museums are missing an important opportunity to meaningfully collect materials produced *by* them (Patterson 2020). This is a missed opportunity, for children are crucial citizens whose knowledge, perspectives, and experiences are valuable. We argue that children’s culture must be collected and preserved during this historic moment and beyond, in ways that attend to the particular circumstances children face as multiply marginalized museum constituents and members of society.

## The Historical Roots of Children’s Exclusion

Within museum settings, collection policies have overwhelmingly privileged the cultural production and material culture of adults. Even children’s museums, who consider young people their target audience, rarely house collections that reflect the lives of children themselves, focusing more typically on ethnographic artifacts, natural history specimens, and general historical materials. Of the few museums that do prioritize the collection of childhood objects, their holdings typically boast an array of toys and playthings, such as those displayed at The Benaki Toy Museum in Athens, the Museum of Childhood in Edinburgh, and the Victoria & Albert Museum of Childhood in London. Historically, few museums have examined the perspectives and lived experiences of young people with explicit focus. However, recent initiatives have begun to do so with newfound vigor. For example, the Museum of New Zealand Te Papa Tongarewa in Auckland began working with seven children and their families in 2012 to “build a collection of objects that represent the lives and experiences of children from different backgrounds growing up in New Zealand” (Museum of New Zealand Te Papa Tongarewa n.d.). Moreover, Australia’s largest public museums organization, Museums Victoria, manages a “Childhood Collection” in which they house objects made by children as well as their “written descriptions of games and play, reflecting their worldviews, experiences, and imaginative lives” and ensuring that “their lives and stories are not subsumed within adult-driven historical narratives” (Tout-Smith 2012). Emerging initiatives such as the nascent Cape Town Museum of Childhood and the Museum of Childhood Ireland are currently building collections with a commitment to documenting both the historical and contemporary social history of children by focusing on children’s heritage and the roles that children play in society. These examples mark a significant change in how children’s objects are collected, demonstrating an interest in capturing young people’s own interpretations of their belongings.

Overwhelmingly, however, the majority of what museums collect pertaining to the lives of children reproduces adult notions of nostalgia and innocence, long critiqued in the field of Childhood Studies (Cunningham 1995; Pascoe 2010). As sociologist Nick Lee (2001) has argued, we often make sense of childhood “through adulthood, interpreting everything children do, or have done to them, in terms of how this will affect their journey toward adulthood” (p. 8). This hegemonic frame of adulthood also casts backward through nostalgic adult (mis)rememberings of how childhood was, and often informs projections of how it should be. Lee argues that this dominant framework not only “mutes” children by casting them as incompetent, irrational, and incomplete *becomings* as opposed to valued human *beings*, but also “grants adults the position of legitimate authorities over them, capable of knowing better than them and speaking more fully on their behalf than they are able to” (Lee 2001, 44). Laurajane Smith has noted similar nostalgic interpretations of childhood foregrounded in heritage sites, both of which are typically cast as “good” and “comforting” (Smith 2013, 115). Just as nostalgia “paint[s] the past as more attractive than it probably ever was,” childhood is often “painted with similar rosy or sentimental hues” (p. 115). Stemming from this convergence, social history exhibitions can spark pleasant childhood memories in visitors, encouraging romanticized perspectives that obscure the complexities of lived experience. Such adult-centric power dynamics are almost always maintained within museum institutions.

Research by museologists and historians such as Sharon Brookshaw and Tory Dawn Swim Inloes further highlight the romanticized layers of sentimentalism permeating the inclusion and framing of childhood objects in museum collections (Brookshaw 2009; Inloes 2014). The most commonly collected items are those that adults remember the most fondly, including toys, clothing, baby items, and photographs (Brookshaw 2009, 375). While these objects help constitute the material culture of childhood, as Brookshaw notes, they are typically things used by children but produced *for* them *by* adults (p. 381). In contrast, the material culture of children, or those objects made, manipulated, and modified by children themselves, is far less frequently collected and displayed in museum spaces (pp. 379–380). This may be attributed to the fact that these productions are often abundant, ephemeral, and fragile in nature, and therefore undervalued and overlooked by both adult family members and museum workers alike. But the lack of children’s objects in museum collections also reflects a broader lack of regard and respect for children’s knowledge, competencies, and contributions. By primarily collecting the material culture of *childhood* (as opposed to the material culture of *children*), Brookshaw argues that museums erase children’s interactions with the objects around them and suppress their unique voices, choosing instead to present a homogenous picture of childhood most often based on (some) adults’ nostalgic constructions. Contemporary collecting related to COVID-19 offers an opportunity to capture a more diverse array of children’s experiences and cultural productions, yet few museums seem well equipped to do so.

## Museum Collecting in the News

**Figure 1.**
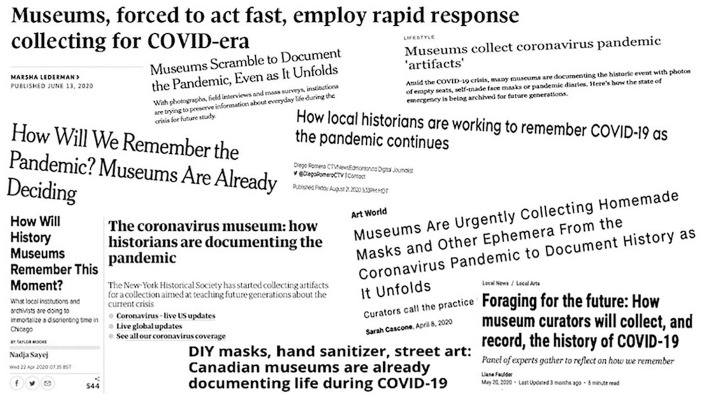
COVID collecting in the news, Screenshot collage by Rebecca Friend, 2020.

As part of the torrent of mass media produced during the crisis, journalists are eagerly commenting on museums’ plans for building their COVID-related collections, while forward-looking institutions have started to collect objects they believe will define the moment (Figure 1). Homemade face masks and medical-grade Personal Protective Equipment (PPE), hand sanitizer produced in local distilleries, toilet paper, and children’s artistic renderings of rainbows have taken positions of prominence in museums’ COVID collections (Haigney 2020; Lederman 2020; Popescu 2020; Sayej 2020). Displayed as messages of hope and gratitude for essential workers in windows across the world, children’s rainbow art has become *the* iconic representation of children during this pandemic. Victoria & Albert Museum of Childhood director Gina Koutsika believes the drawings are valuable because they bring “cheer to others in what is a disconcerting and potentially frightening time” (Brown 2020). On the one hand, the signs are a form of social engagement and action, whose varied mediums and messaging provide a platform for children’s voices and artistry. But the singular focus on this specific type of cultural production, and the emphasis on the cheerful and uplifting nature of children’s rainbow art, ultimately overshadows the diverse lived experiences of young people grappling with COVID-19’s innumerable impacts on their daily lives.

Children’s window rainbows reproduce the dominant trope of childhood innocence (Garlen 2020), a construct long celebrated despite being an exclusionary form of social practice (Garlen 2019) that essentializes and obscures the highly variable and complex nature of children’s experiences. Specifically identified as a collecting priority by institutions like the New York Historical Society, the Museum of London, the Victoria & Albert Museum of Childhood, and the Portsmouth City Council’s Museums and Archives (Cascone 2020; Portsmouth City Council 2020; Thompson 2020), the rainbow sign has become a ubiquitous form of children’s pandemic productions. While the rainbow drawings do represent both a trending social phenomenon and a universal symbol for hope in this time of crisis, prioritizing their collection over more intimate and varied explorations of young people’s unique viewpoints further entrenches a reified, romanticized, and simplistic narrative of childhood in the time of COVID.

## Collecting Children’s COVID Culture in Practice

Despite the museum sector’s unprecedented efforts to prompt children’s productions at home through online museum programming and the encouragement of online sharing via participatory hashtags such as #ChildrensMuseumsAtHome, #MuseumAtHome, and #MuseumWindow, little attention or resources have been devoted to collecting these creations. Moreover, questions concerning what will become of the vast sea of digital content posted on social media sites such as Facebook, Instagram, Twitter, and TikTok have remained unanswered. Nevertheless, some museums have explicitly sought out young people’s perspectives and cultural productions in their newly-minted collecting initiatives, serving as models for ensuring children and youth are not overlooked in the race to remember the pandemic. For instance, Ontario’s Huron County Museum is collecting personal stories from local residents digitally, through mail, or in person once the museum resumes its operations, and prompt questions feature a section for “children, or anyone young at heart” that asks what they miss, how they are coping with distance education, and what life at home is like (Huron County 2020).

Other museums in Ontario have shown prescience in producing linked programming and collecting initiatives that prompt children and youth to record their experiences and perspectives in COVID-themed journals to later be collected and preserved. Museum Windsor’s journaling initiative, created through a partnership with the Windsor Public Library, includes questions specifically for students that touch on how their lives have changed, what anxieties they are experiencing, and what new hobbies they have developed (City of Windsor | Museum Windsor 2020). Similarly, Niagara-on-the-Lake Museum’s junior and intermediate workbooks feature an array of documentary activities for young people that include writing and illustrating a book detailing their days at home, creating COVID-19 themed comic strips, starting a scrapbook, conducting an interview with their parents, making their own memes, and writing letters to help inform future historians what mattered most to them during this time (Niagara-on-the-Lake Museum n.d.). Also featured in the workbooks are calls to record any “kindness projects” they are a part of that help uplift community members. At the Children’s Museum of Pittsburgh and Lennox & Addington Museum & Archives, collecting plans are documenting young people’s participation in similarly inspiring “Kindness Initiatives” happening in their local areas (Children’s Museum Pittsburgh 2020; County of Lennox & Addington 2020).

One of the most ambitious efforts to collect children’s cultural productions and perspectives may be the Museum of Childhood Ireland’s expansive “Project 2020 Together/Le Chéile.” The nascent museum states its commitment to a child-centered philosophy that solicits, collects, and exhibits children’s perspectives and cultural production through multiple projects. Launched on June 4, 2020 to document children’s perspectives on pandemic life, “Project 2020 Together/Le Chéile” exhibits children’s art, photography, and writing from around the world. Organized under six themes (family and home, my friends, my toys and games, places I miss the most, my favorite book, sports at home, and how I feel), the children’s creations are currently displayed online. Lamentably, the museum removes children from the submission process almost entirely, asking only that their parents or carers confirm that the child has given permission for their work to be shared. Children’s real names, or the names of their acquaintances, are not allowed to be included in their creations, yet their first names, age, and location will be attributed to their work. The museum also notes that frames designed by (adult) artist Carol Ann Tracey will be added to the online and eventual physical exhibitions “to compliment the children’s work” (Museum of Childhood Ireland 2020). These small but significant details reinforce adults’ positions as gatekeepers and undermine the otherwise progressive approach of the museum to empowering children. What is most notable about these virtual exhibits is the wide range of formats and contents of the submissions themselves. Different geographic and cultural contexts are represented, along with feelings of fear, boredom, sadness, outrage, playfulness, nostalgia, and hope. The museum is also collecting copies of children’s letters in their Gel Project letter writing initiative, and artifact suggestions from Irish children documenting the pandemic in Ireland through the “Memory Boxes” project.

## Epistemological Challenges of Collecting Children’s Materials

While the aforementioned examples do the work of encouraging young people to feel included in COVID-centered narratives and transcend the standard request for rainbow drawings, their formats nevertheless restrict children’s capacity to take charge of the form, content, and exhibition of what they wish to see remembered, highlighting some of the epistemological challenges involved in collecting children’s material and intangible culture. With few exceptions, current collection calls targeting children leave little room for their own spontaneity and creativity, offering at best templates developed and produced by adults that children are invited to fill in. They also disproportionately draw from Western, middle-class, and White cultural traditions that privilege European artistic genres, written versus oral storytelling, and Western musical heritage (as opposed to genres such as rap, throat-singing, and spoken word, for instance). The perduring dominance of White culture, people, and perspectives reflects long-standing forms of inequality stemming from historically entrenched racism and other forms of elitism firmly embedded in museum practice (Cocotle 2019; Heller 2017; McCambridge 2017; Moore 2020; Randal 2020). Arranged in prescribed, culturally-specific formats, museums’ questions and fill-in-the-blank boxes set standards for young people to follow, ultimately directing the types of materials and the content they will be collecting.

A further epistemological weakness of the template approach stems from the fact that these templates are not only designed, produced, and collected by adults, but are also interpreted by them almost always independently from children. Such an approach perpetuates a dominant paradigm that privileges adult knowledge about children while eclipsing children’s own expert knowledge about themselves. It also perpetuates Eurocentric, patriarchal, hetero-centrist, classist and in other ways exclusive and partial knowledge production through an implicit (and false) claim to universalism, obscuring variation and complexity across locality and within and among individuals. This epistemic inequality renders adults—and museum practitioners who are charged with documenting, preserving, and interpreting knowledge and culture— unable to fully recognize children’s knowledge. It obscures children’s agency and interiority, and subordinates their own expertise on themselves. This is especially problematic given that, as Lansdown (2005) concludes, “it is increasingly clear. . . that adults consistently underestimate children’s capacities. . . [and t]his misconception takes different forms in different cultural contexts” (pp. 30–31). The dominant narratives and approaches being utilized by museums—including the more progressive initiatives that acknowledge children at all—limit what is known and knowable about children, but also about society, and the COVID-19 pandemic museums are increasingly seeking to document.

## Ethics of COVID Collecting and Intersections With Ethical Dilemmas in Childhood Studies

The epistemological challenges that come with collecting children’s culture during the COVID crisis are deeply intertwined with a host of ethical challenges. What is collected—and collectible— affects what is known and knowable, for museum collections and archives constitute a significant (if partial) record for future generations. Dominant frames and those who work within them do not typically recognize their own incompleteness, but nevertheless reproduce flawed and partial knowledge. A plurality of perspectives on the pandemic should include those from society’s youngest members and reflect the diversity of experiences, contexts, and complexities of childhood across the world. Multiply marginalized voices of poor, non-Western, and non-White children must be recognized, collected, and preserved as well as those from demographic groups more often engaged by museums.

Informed consent is particularly important and complex as it pertains to collecting contributions from children. Until relatively recently, many scholars deferred issues of consent to a child’s parent/s or legal guardian/s, reflecting the legalistic frameworks that empower only adults to consent (James and James 2008). But child advocates and scholars in the field of Childhood Studies have insisted that children should also give their informed consent before participating in research (Albon 2020). This requires that they understand and agree to the implications of the research, or in this case agree to donate to a collection that may be used for future research or display. Museums must also carefully think through who is allowed to submit children’s cultural productions to collections, and under what conditions.

All too often, ethical protocols, policies, and laws that seek to protect children from exploitation and harm can also cut them off from forms of public engagement, obscure and even erase them from various archives, and silence their voices and contributions. It is incredibly complicated for museums to share photographs of children and their cultural production with the public, particularly online. While it is important to protect children from harm, such efforts can also undermine children’s right to express themselves, to access information, and to participate freely in cultural life and the arts, as enshrined in the UN Convention on the Rights of the Child, which came into force in 1990 (United Nations 1989). It is important, particularly in cases of artistic work and writing, that children be granted ownership over their work and be allowed to choose how their contributions will be presented and attributed. While some may wish to have anonymity, others may justifiably feel that they want their name, in part or in full, to be credited.

## Conclusion

**Figure 2.**
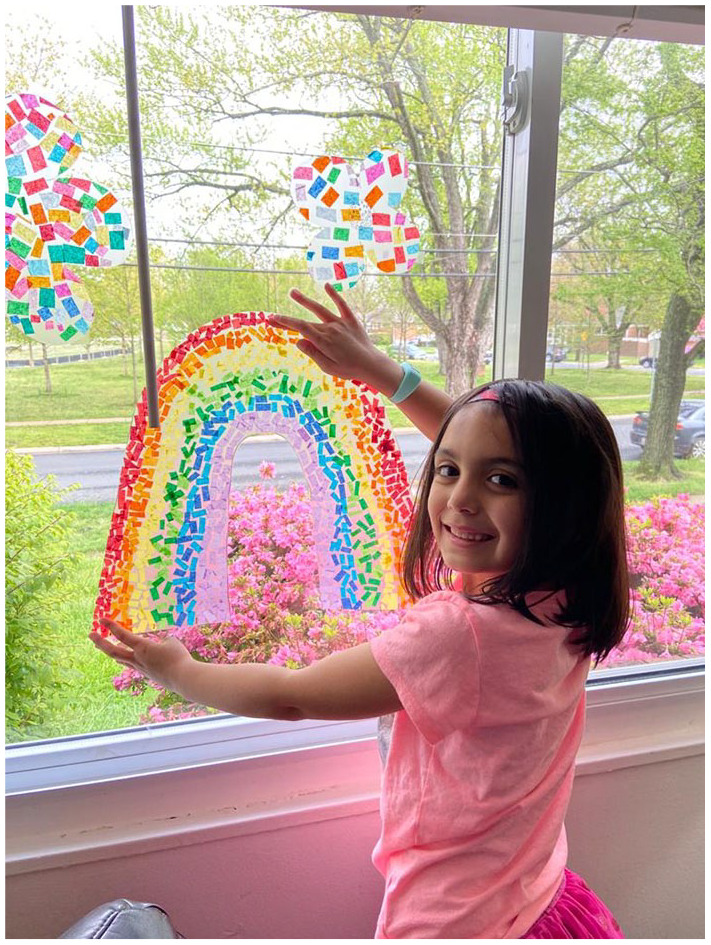
Six-year-old Kaylen Phillips proudly displays the rainbow she made in the front window of her home. *Note*. Photo credit: Kathleen Posey Phillips, April 24, 2020.

As the rush to contain the COVID-19 virus gives way to new norms of practice and social engagement, museums will continue to provide important leadership in collecting, commemorating, and (re)constituting community during and after the crisis. While the move to rapid-response and online forms of collecting have in many ways helped to increase public participation, it would appear that those whose voices have been historically marginalized from museums continue to be excluded now. This certainly pertains to children, despite the proliferation of activities being produced for them. There is a pressing need to decenter adult-centered paradigms that invite children to imitate adults, answer their questions, and conform to their frameworks. Recent collecting initiatives rarely recognize children as important contributors to their COVID-collections, with the exception of an already consolidated, singular, and sentimentalized artifact: the window rainbow sign. And yet, these signs contain multitudes, especially for the children who produced them: they are beautiful, poignant, powerful, playful, expressive, and inspiring (Figure 2). They help show that children are knowledgeable and contributing members of society. But they have also become reified in ways that silence other children, and other forms of children’s cultural production. In the absence of the kind of contextualized evidence that a wider range of individual children can provide about their interior worlds, experiences, and perspectives, COVID collecting campaigns miss a crucial opportunity to preserve and learn from children, for they too make meaning, and make history.
